# Ammonia plasma-treated electrospun polyacrylonitryle nanofibrous membrane: the robust substrate for protein immobilization through glutaraldhyde coupling chemistry for biosensor application

**DOI:** 10.1038/s41598-017-10040-7

**Published:** 2017-08-25

**Authors:** Matin Mahmoudifard, Masoud Soleimani, Manuchehr Vossoughi

**Affiliations:** 1 Institute for Nanoscience and Nanotechnology, Sharif University of Technology, Tehran, Iran; 2 Stem Cell Technology Research Center, 19977-75555, Tehran, Iran; 3 Department of Haematology, Faculty of Medical Sciences, Tarbiat Modares University, Tehran, Iran; 4 Chemical and Petroleum Engineering Department, Sharif University of Technology, Tehran, Iran

## Abstract

The surface of polyacrylonitrile electrospun nanofibrous membrane (PAN NFM) was aminated by the ammonia plasma treatment. The content of amine groups has been estimated for different time of plasma treatment. The newly generated amine groups were successfully activated by glutaraldehyde (Ga) for the covalent attachment of the protein molecules on the NFM surface. Bio-functionalization of ammonia plasma treated PAN NFM was carried out by the primary antibodies (Ab) immobilization as a protein model through Ga coupling chemistry. For comparison, the immobilization of Ab was also performed through physical interactions. Attenuated total reflection-fourier transform infrared spectroscopy (ATR-FTIR) was used for the characterization of surface functional groups of PAN NFM after different modifications. The surface morphology of the NFM after immobilization was characterized using scanning electron microscope (SEM). The efficacy of Ab immobilization was estimated by enzyme-linked immuno sorbent assay (ELISA) method. X- Ray photoelectron spectroscopy (XPS) was performed to confirm the covalent immobilization of Ab on the modified PAN NFM. Results show that ammonia plasma treatment effectively increased the amount of Ab immobilization through Ga coupling chemistry. Our findings suggest that this is a versatile model for the preparation of stable bio-functionalized NFM which is applicable in different field of biomedical science.

## Introduction

Protein biomarkers detection using a biosensor devices is the standard method for early detection of diseases such as cancer, cardiac vascular attacks^1, 2^, alzheimer^3^ and infection^4^. Hence, antibody (Ab) or antigen immobilization onto a solid surface is a key parameter for the fabrication of most immune-sensors, including ELISA^5^, 2-D western blotting^6^, 2-D gel electrophoresis^7^ mass spectroscopy^8^. Up to now, extensively attention have been established to proposed biosensor for biomedical, environmental and analytical assays which led to the fabrication of some commercially available, compact and point of care biosensor devices^9^ and still this subject attract special attention of many health care researchers around the world.

ELISA technique has been widely applied for the early detection of diseases based on proteins biomarker^10^ and providing a low cost and detection limit with high sensitivity and selectivity.

Typically, ELISA is carried out in conventional 96 wells plate and they are mostly fabricated from polymers with poor hybridization site for protein immobilization like polystyrene (PS). In such plates protein immobilization occur in the form of physical adsorption. In most of cases, physical immobilization may lead to partial or complete loss of protein activity, due to structural deformation and random orientation. Therefore, oriented immobilization is more preferred for the improvement of immune-sensor performance^11^. Oriented immobilization of the protein means that the capture proteins are immobilized in such a way that their recognition sites are uniformly exposed to the sample solution^12^. For example, about Ab immobilization, an ideal orientation of the Ab is end-on form which means the Fc region of Ab adsorbs onto the solid surface and the Fabs region orient toward the analyte solution (end-on)^9^. As it is mentioned, commonly used immobilization methods namely, physical absorption technique suffers from non-specific interaction which may lead to desorption of the biomolecule during the intensive washing steps^13–15^. Thus, controlled oriented covalent attachment of antibodies is more preferred compared with random one in ELISA type immune-sensor as it helps to achieve better homogeneity in Ab immobilization. However, creating covalent immobilization of biomolecules on the non-functionalized surfaces including PS is impossible. For example it was proposed^16^ to graft methacrylate on PS micro-titer plate to enhance proteins immobilization but it was reported that such system suffer from poor reproducibility. Therefore, the creation of synthesized or modified functional groups on the solid surfaces could be applied for ELISA technique. For example, up to now, surface modified Nylon, PS, Dacron and Polyacrylonitrile (PAN) are being proposed for the development of a reliable substrate for biomolecules attachment^17^.

The development of nano-structured materials, such as NFM with unique biochemical and biophysical properties, could be a promising candidate for the fabrication of biosensors with higher sensitivity, rapid response and low cost compared to conventional one. PAN is one of the most important spin-able polymers with many desirable properties such as abrasion resistance, solvent resistance, thermal, chemical and mechanical stability, high tensile strength and good insect resistance^18–20^. Nowadays, PAN membranes find many attractive applications in the vast areas of sensor, composites, protective clothing, gas separation, and biomedical applications^21–23^. Moreover, surface modification of PAN fibers have been opened up new window for the immobilization of different enzymes and protein for biomedical applications^22, 24^. The surface modification of polymers can be classified into two general categories including wet-chemical process or dry process which occurs at a gas-solid interface like plasma discharge treatment. For example, the formation of amine groups on PAN surfaces is not only possible by an ammonia plasma treatment but also possible by the chemical reduction of nitrile groups of the PAN through lithium aluminum hydride^25^. S. Jain *et al*. reported surface modification of the PAN fibers in order to introduce primary amine grope by reduction with lithium aluminum hydride^17^. The objective of this study was to evaluate the effect of amine content on immobilization and stability of the Ab immobilization on the surface of PAN fiber.

To the best of our knowledge, there is no literature about Ab immobilization on ammonia plasma treated electrospun PAN nanofiber. For this purpose, here in this study, amine functionality was induced on the PAN NFM surface by ammonia plasma treatment. The content of plasma produced amine group on the surface of PAN NFM was determined and the best plasma time was chosen for optimum amine content on the surface of membrane. The feasibility of Ab immobilization through Ga coupling chemistry on the ammonia plasma treated PAN NFM was assessed.

## Experimental

### Materials

PAN powder (Mw = 4 * 10^5^ g/mol) was supplied by Polyacryl Co. (Isfahan, Iran) and N,N dimethylformamide (DMF) was obtained from Merck, respectively, as polymer and solvent. Ga and Tween-20 were purchased from Sigma–Aldrich. TMB substrate solution was obtained from Razi Biotech. Bovine serum albumin (BSA) and sulfuric acid (H2SO4) were supplied by Merck. Here, we used intravenous immunoglobulin (IVIG) as a protein model for immobilization on the NFM surface. IVIGs are sterile, purified IgG products manufactured from pooled human plasma and typically contain more than 95% unmodified IgG, which has intact Fc-dependent effector functions. Secondary Ab mouse anti-human IgG-HRP conjugated was purchased from Abcam.

### NFM preparation and ammonia plasma treatment

The solution of PAN was prepared by dissolving 5 w/v% of PAN powder in DMF via magnetic stirrer at 40 °C for 24 h. The prepared solution was poured to a plastic syringe with a 22 G needle tip (inner diameter: 0.4 mm and length: 34 mm). The flow rate of the polymer solutions was kept to 0.3 ml/h, The distance between needle and collector was adjusted to 15 cm. High voltage of 16 kV was applied between the nozzle and a rotating collector (take-up speed was 250 rpm) to force the solution droplet from the needle to fabricate fine fibers with the nanometer diameter on the collector. Surface modification of the fabricated PAN NFM was carried out through ammonia plasma treatment by a low frequency plasma generator with a cylindrical chamber (24 l) that all around the glass chamber were covered with cylindrical electrodes (Diener plasma system, Germany). As we know, when plasma systems run on a low plasma frequency they create longer wavelengths which give ions a large amount of kinetic energy. With a lower plasma frequency there is a higher ion density so the sample position in this space everywhere will have a similar result. But we placed sample in middle of chamber and electrodes anyway. In such a low pressure system, chamber pressure is set instead of the gas flow rate. Ammonia gas was introduced into the reaction chamber at 0.4 mbar and glow discharge was flowed for desire time. The power was set 30 W and the process time was 2, 4, 6, 8, 10, 12 min.

### Characterization study of NFM

The surface morphology of PAN NFM were examined through scanning electron microscopy (SEM) (Seron, AIS2300C, Korea) after gold spattering on membrane. Moreover, SEM was also used to further evaluate how the morphology of non-plasma and plasma-treated NFM will be after incubation with gataraldehyd and Ab immobilization. Moreover, ATR-FTIR (Thermo Nicolet model: NEXUS 670, USA) was used to investigate the chemical characteristic of the membrane after plasma treatment, incubation with Ga and Ab immobilization.

### Determination of amine group content of PAN NFM produced by plasma treatment

Primary amine content of plasma-treated PAN NFM was evaluated by methyl orange. Methyl orange is an acidic dye which can bind to the amines group of polymeric substrates. The dye can be detached again by bases solution^25^. Thus, this property of methyl orange can be used to determine the content of amine groups created on the NFM surfaces by plasma treatment.

Electrospun PAN NFMs which prepared by plasma modification technique under various time were incubated with the acidic methyl orange solution (0.05% methyl orange in a 0.1 M solution of sodium dihydrogen phosphate (Merck) in water, pH: 4.8) for 2 h. After rinsing with distilled water, the methyl orange-colored membranes was incubated by 5 ml of a 0.1 M potassium carbonate solution to remove all of the absorbed methyl orange from membranes and then the leached dye concentration was determined photometrically at 460 nm.

### Covalent immobilization of antibodies onto modified PAN NFM

Three types of substrates, i.e. non-plasma, plasma-treated PAN NFMs and 96-well micro-titer plate were used for immunoassay procedure. Plasma-treated and non-treated NFMs with the thickness of 75 μm were punched in order to fit the wells of 96-well plates (diameter of 0.5 cm). First, the activation of plasma treated and non-treated PAN NFMs were carried out using incubation in 4% aqueous solution of Ga (pH: 8.5) at 4 ^◦^C for 3 h. The electrospun NFMs were thoroughly washed with Tween/PBS (pH: 7.2) and water to remove excess of Ga. Ab capturing efficiency on different surfaces was evaluated by means of ELISA technique. For this purpose, IVIG Ab was used for immobilization on different test surfaces and polyclonal mouse anti-human IgG-HRP conjugated was used for detection of immobilized Ab. Moreover, physical immobilization of Ab was also examined in order to compare the efficiency of covalent capturing of Ab through Ga on membranes with physical one. The reagents used for our ELISA process were as follows: primary Ab 20 μg/ml in PBS buffer, pH: 7.2, BSA 2 wt% in PBS buffer, pH: 7.2 as blocking agent, HRP-conjugated secondary Ab in BSA 3 wt% in PBS buffer, pH: 7.2 buffer (1:8000, pH: 7.2), TMB substrate as signal generating agent and sulfuric acid 1 N as stop reagent. Ab immobilization was conducted by pipetting primary Ab (100 μl, 20 μg/ml) into each well containing plasma and non-plasma NFM and incubation for 1 h at RT. The wells were washed 3 times by wash buffer (PBS/tween20 (0.05%v/v). Blocking treatment was carried out by incubation of each well with 100 μl BSA 2% for 1 h in RT. After removing the BSA solution secondary Ab capturing was conducted by incubation of each well with 100 μl secondary Ab for 1 h at RT. Each well were washed out 5 times with wash buffer for thoroughly removal of unbound antibodies, peroxidase activity of the bound secondary Ab on the fiber was measured by the conversion of colorless TMB substrate to a blue colored product immediately after 20 min. To stop enzymatic reaction, 100 μl H2SO4 solution was added to each well to generate final signal. Then 100 μl solution of each well was transferred to beneath well (because the membrane existence in well causes error in signal reading by machine). The absorbance at 450 nm was recorded using a multi-plate reader (Biotec, ELX800, USA). It should be noticed that control membranes used in this study were plasma and non-plasma treated membranes which only exposed to the secondary Ab and all the result reported after subtraction of control value from sample value.

### Visualization of Ab immobilization efficacy on PAN electrospun nanofiber

In order to compare the binding capacity of Ab immobilization on plasma and non-plasma treated PAN NFMs through different immobilization techniques, with and without Ga following experiment was also designed. After performing the immobilization of primary Ab and blocking steps, all membranes were incubated for 1 h with 1 μl/ml FITC-labeled human IgG solution at room temperature. The NFMs were then washed thoroughly five times with wash buffer to remove excess FITC-labeled Ab and analyzed for fluorescence intensity by means of fluorescent microscope (Nikon, Eclipse TE2000-S, Japan).

### Stability test of immobilized antibodies on PES NFM

A set of experiments were designed to assess the stability of immobilized antibodies from denaturation during storage period. For this purpose, after immobilization of primary Ab on different substrates (as mentioned above) with and without Ga as a coupling agent, all the wells were washed three times and allowed to dry at RT. After drying, all plates were placed in sealed plastic containing silica gel desiccant. Three identical sets of specimens were prepared and stored at 4 °C. The rest of the ELISA procedure including blocking step and signal generating step were carried out after passing 1, 2 and 3 months.

### Antibody binding capacity of nanofibrous membrane through two different strategies

The capacity of PAN NFM for Ab immobilization on their surface through two different strategies was evaluated by the calculation of difference in the amount of the adsorbed BSA before and after of antibody immobilization on the NFM. For this purpose, the NFM were incubated for 1 h in blocking solution (3% BSA in PBS) before and after antibody immobilization. After incubations, the concentrations of leached BSA in the supernatant were measured by UV-visible spectroscopy (Perkin Elmer Company) at 280 nm. The concentration difference of leached BSA before and after Ab immobilization was calculated to assess the amount of immobilized antibody on the surface of the NFM.

### X-ray photoelectron spectroscopy (XPS)

X-ray photoelectron spectroscopy (XPS) equipped with a monochromatic Al-Kα X-ray source at energy of 1486.6 eV was utilized as a complimentary method to quantify the chemical compositions of PAN NFM, PAN-NH2, PAN-NH2-Ga and PAN-NH2-Ga-Ab. The analyzer (Specs EA 10 Plus) was in the shape of hemispherical. The base pressure in the analytical chamber was maintained at 10^−8^ Torr or lower during each measurement. All measurements were made at a photoelectron take off angle of 90°. The samples were mounted on the standard sample holder by means of adhesive tapes. All binding energies (BEs) were referenced to the C1s hydrocarbon peak at 284.7 eV as a reference energy point. Spectra were analyzed using SDP software (version 4.1) with variation of Gaussian (100–80%) ± Lorentzian (0–20%) profiles for each component to obtain the best fitting.

## Results and Discussion

**Figure 1:**
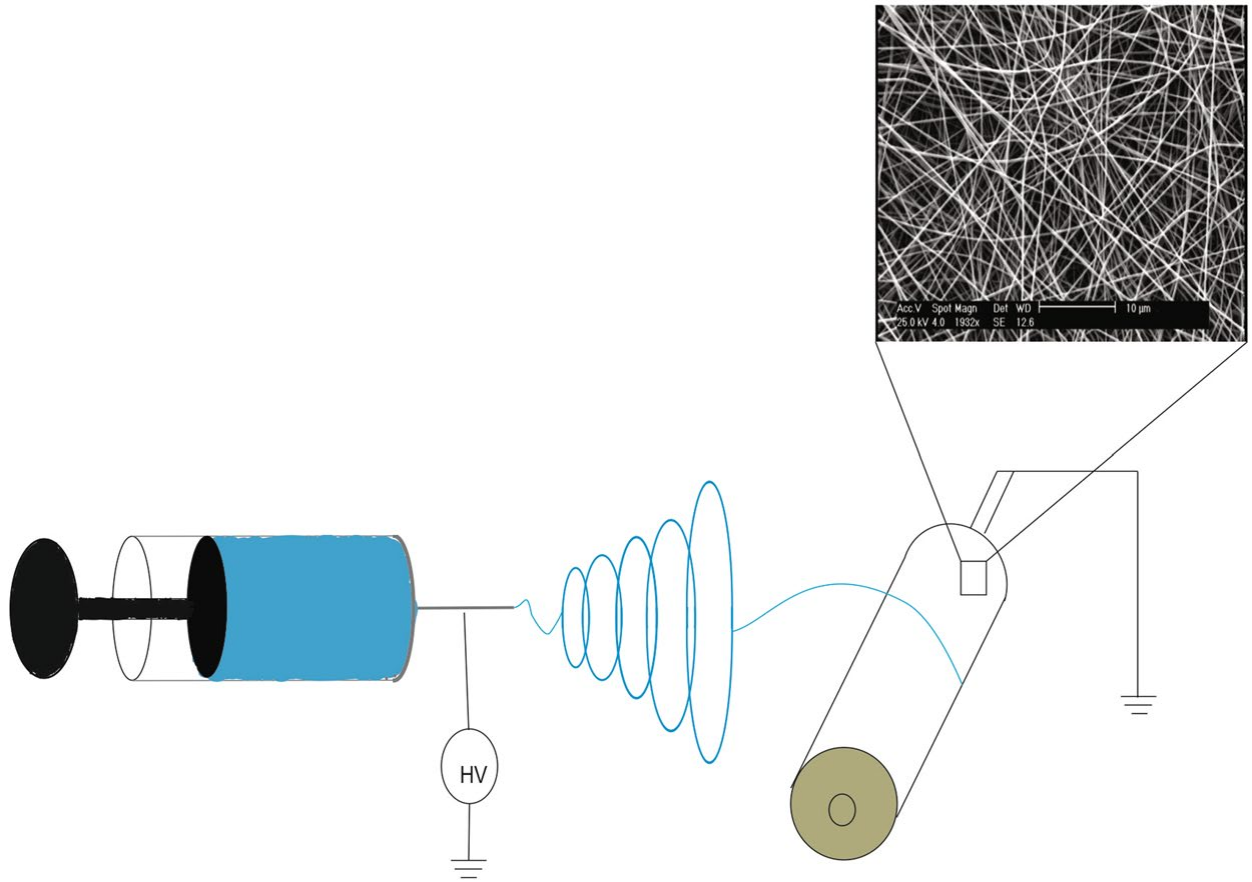
Electrospinning system and the morphology of resulting NFM.

All the required conditions for electrospinning of PAN such as voltage, distance between needle tip and collector, flow rate were optimized to obtain uniform and bead-less NFM. The resulting morphology of the electrospun NFM was shown in Fig. 1. The surface modification of PAN NFM was carried out through ammonia plasma treatment.

**Figure 2:**
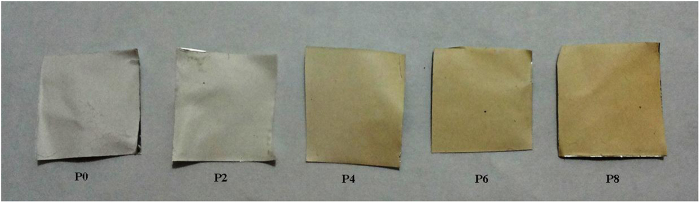
Image of plasma treated PAN NFMs with different time duration.

**Figure 3:**
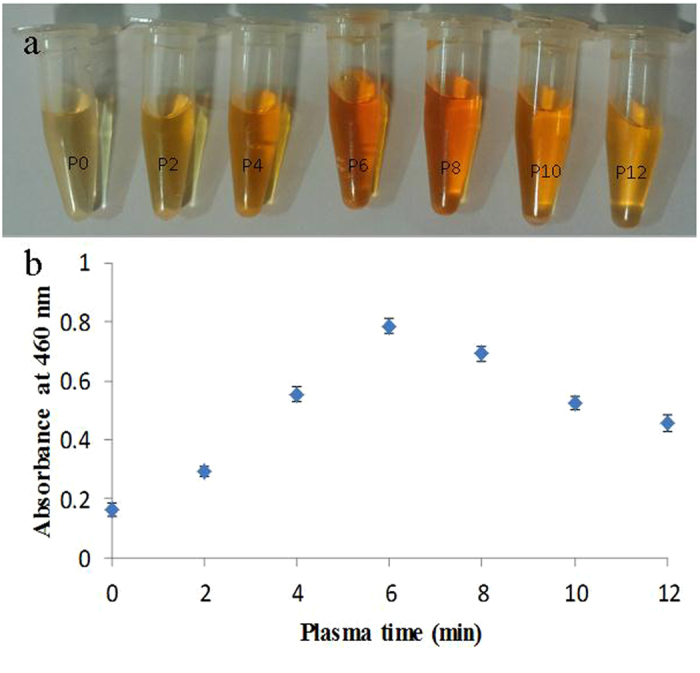
(**a**) The concentration change of eluted methyl orang from PAN NFM with different plasma treatment time duration (**b**) absorbance chances of eluted methyl orange from ammonia plasma treated PAN electrospun NFM with different plasma duration.

Ammonia fragmented in glow discharge and fragment can vary from NH2 and NH radicals to nitrogen and hydrogen. It was reported depending on gas pressure, power, plasma time and so on there is always a distribution of different gas fragments, which affects the composition of top layer of re surface^25^. In addition to NH2 incorporation to the surface, the nitrile groups of the PAN can be reduced by the hydrogen fragments exist in the plasma. From Fig. 2 it can be observed that after the amination, the color of membrane tends to change from white to yellow and the yellowness intensity increased with the advancement of plasma time as result of increasing conversion of nitrile to amino groups of PAN. Similar observation also reported in another study in which PAN fibers were reduced through chemical reaction of lithium aluminum hydride^17^. The amine group content of membrane was visualized by Methyl orange. Figure 3 illustrates the concentration change of eluted methyl orang from PAN NFM with different plasma treatment time. As it is observe the maximum concentration of eluted methyl orange obtained when 8 min plasma treatment carried out on the membrane. With increasing plasma time it seems that the content of amine group tends to decrease. This means that the modified PAN membrane surface is changed by collisions of plasma particles at higher plasma time. It known that bombardment of the polymer surface by plasma particles in the intense manner (higher exposure time, higher power and so on) cause the polymer surface start to etch and degrade, in this case plasma composition around the surface change and cause the efficacy of surface functionalization is decreased^26^. Another possible phenomenon which could occurred in higher plasma time is crosslinking between two proxy functional group exist on the surface. This phenomenon also cause the number of created functional group decrease.

**Figure 4:**
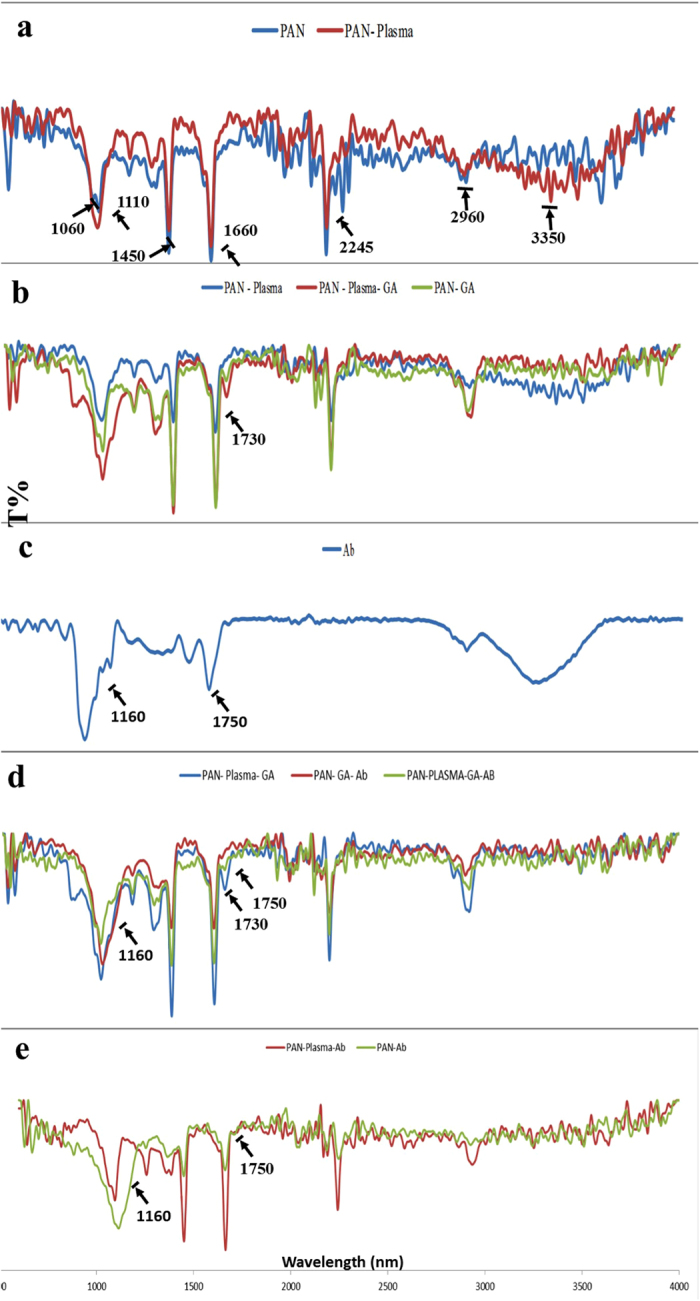
ATR-FTIR spectra of (**a**) unmodified and plasma-treated PAN NFM (**b**) plasma-treated PAN NFM and Ga- activated PAN NFM (**c**) antibody (**d**) Ga- activated PAN NFM and antibody immobilized PAN NFM (**e**) Ab immoibilization through physical interaction on plasma and non- plasma PAN NFM.

The FTIR spectrum of PAN NFMs with different modifications present in Fig. 4. Figure 4a showed characteristic peaks of un-modified PAN NFM at 2245 cm^−1^ which is assigned to stretching vibration of nitrile group, strong 1450 cm^−1^ of C-H related to in-plane deformation vibration of CH2 groups. The characteristic absorption peaks at 1110 and 1060 cm^−1^ represent nitrile stretching and bending, respectively. The peak at 2960 cm^−1^ corresponds to C-H stretching vibration. A broad band at 3350 cm^−1^ appeared after ammonia plasma treatment which is attributed to the N-H stretching vibration as a result of the primary amine group formation. Moreover, the peak intensity of 2245 cm^−1^ corresponding to the nitrile group decreased in magnitude after plasma treatment indicating nitrile group reduction to primary amine. After Ga-treatment, the absorption peak of free carbonyl group of Ga at 1730 cm^−1^ was clearly observed^17^ about both non- plasma and plasma treated PAN NFM. However, as it is observe the intensity of this peak is significantly higher with plasma treated PAN NFM (Fig. 4(b)). The spectra of Ab immobilized PAN NFM through Ga coupling chemistry represent distinct absorption bands at 1160 and 1750 cm^−1^ different from that of Ga treated PAN NFM (Fig. 4(d)), which are also appeared in the spectrum of Ab immobilization through physical adsorptions on plasma and non- plasma PAN NFM given in Fig. 4(e) for comparison. Moreover, as it is observed with antibody immobilization, the intensity of peak in 1730 which is related to aldehyde groups of GA start to reduce indicating the creation of new covalent bonding by antibody molecules. Other peaks of Ab were overlapped with Ga-treated PAN NFM peaks.

### Antibody immobilization

**Figure 5:**
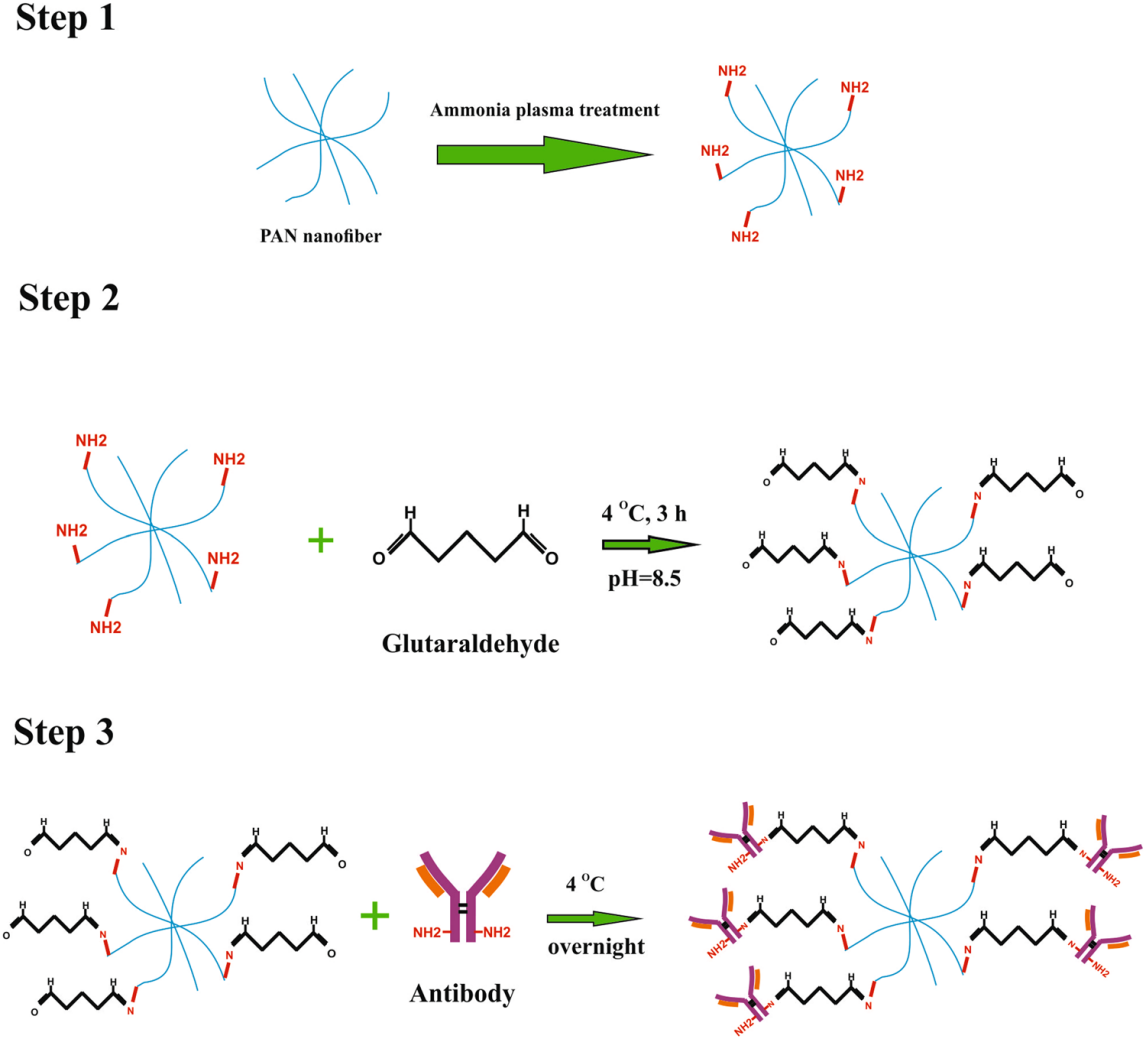
Activation of the plasma-treated PAN NFM fibers with glutaraldehyde and immobilization of antibody on the activated NFM.

After activation of plasma treated PAN with excess of bi-functional Ga, as it is schematically illustrated in Fig. 5, the primary amine groups of the PAN start to react with one of the aldehyde groups of Ga to form the imine linkage. On the other hand, the free aldehyde group of Ga has ability to bind covalently to the amine groups of the antibodies, providing a stable immobilization of antibodies on the Ga-activated PAN NFM.

**Figure 6:**
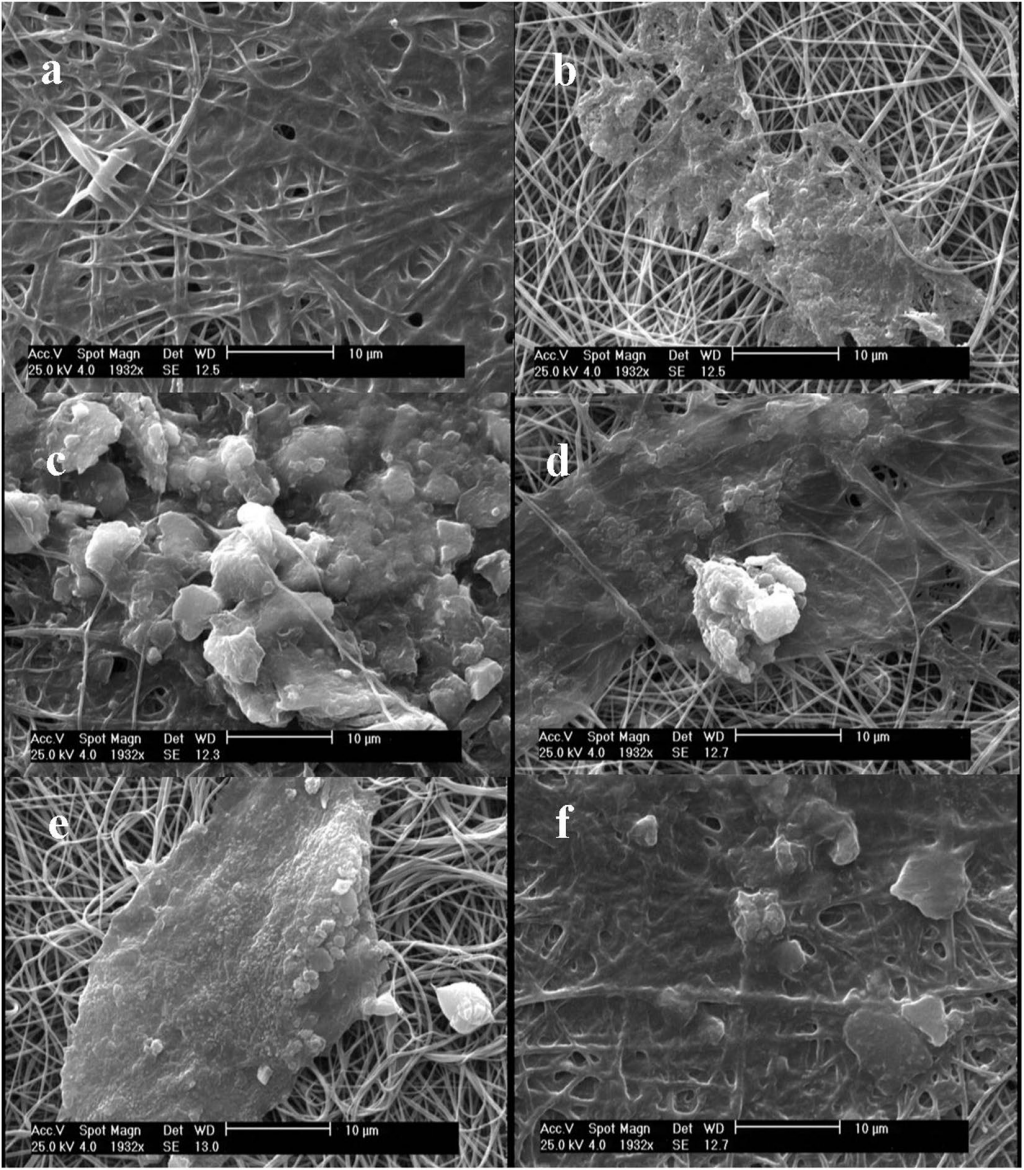
Scanning electron microscopy images of (**a**) Ga coating on ammonia plasma-treated PAN NFM (**b**) Ga coating on non-plasma treated PAN NFM (**c**) 20 μg/ml Ab immobilization through Ga coupling chemistry on plasma-treated PAN NFM, 20 μg/ml Ab immobilization through Ga coupling chemistry on non-plasma treated PAN NFM (**e**) 20 μg/ml Ab immobilization through physical interactions on plasma treated PAN NFM (**e**) 20 μg/ml Ab immobilization through physical interactions on non- plasma treated PAN NFM.

Figure 6 depicts SEM images of PAN NFM with different treatment. When ammonia plasma-treated NFM activated with the Ga coupling agent, a uniform coating is formed on the membrane surface (Fig. 6a). On the other hand, most of Ga is washed out from non-plasma PAN NFM (Fig. 6b) because Ga physically adsorbed on it through weak forces. It is also observed from Fig. 6c and d that higher amount of Ab will be attached on ammonia plasma treated PAN NFM through Ga coupling strategy compared to other groups. About physical strategy, antibodies molecules tend to attached more on non- plasma treated surface compared to plasma treated one, may due to the difference between inherent features of Ab molecules and plasma treated membrane. The more hydrophilic nature of plasma treated NFM (it was observed that after plasma treatment of PAN NFM, it becomes hydrophil and water contact angle reduces from 88 ± 5 to near zero) and hydrophobic feature of Ab molecules causes relative repellency between membrane surface and Ab molecules. On the ammonia plasma treated PAN NFM, Ab molecules enjoy to shrink to bundles (Fig. 6e) compared to Ab immobilization through physical interactions on non-plasma treated NFM which expand themselves and form a dense coating on the membrane surface (Fig. 6e and f). The same observation also reported in other studies^27–29^.

**Figure 7:**
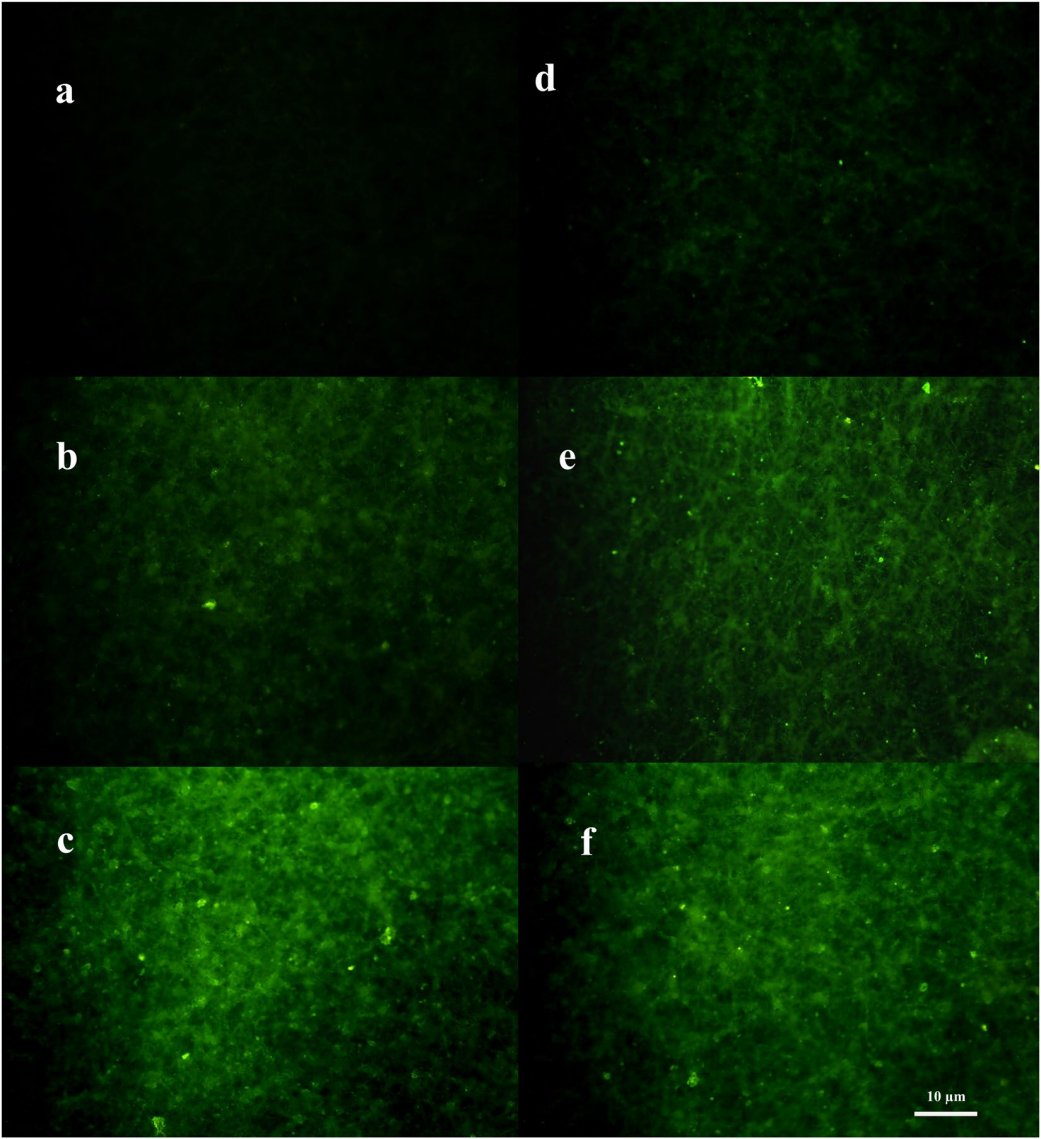
Fluorescence intensity of FITC-labeled anti-human IgG Ab incubated on (**a**) ammonia plasma- treated PAN NFM (**b**) Ab immobilized plasma treated PAN NFM through physical interactions (**c**) Ab immobilized plasma treated PAN NFM through Ga coupling (**d**) non-plasma PAN NFM (**b**) Ab immobilized non-plasma PAN NFM through physical interactions (**c**) Ab immobilized non-plasma PAN NFM through Ga coupling. (FITC-labeled anti-human IgG Ab immobilization was performed after BSA incubation).

In order to visualize the efficacy of the Ab immobilization on the plasma treated NFM through two different mechanisms, namely physical adsorption and Ga coupling chemistry, fluorescent microscopy were utilized by virtue of FITC tagged secondary Ab (Fig. 7). As it is observed, the fluorescent intensity of control group (Membrane without primary antibodies, was approximately zero for plasma treated nanofibrous membrane (Fig. 7a). Compared to physically immobilized antibodies (Fig. 7b), the highest intensity was observed with Ab immobilized membrane through the Ga coupling chemistry about plasma treated memberane (Fig. 7c). On the other hand, on the non-plasma membrane, a little false positive signal is observed about control group that it may because of hydrophobic nature of membrane which tend to adsorb secondary Ab through physical adsorption (Fig. 7d). The same observation was also reported elsewhere^30^. For non- plasma membrane Again higher fluorescent intensity obtained for antibody immobilization through Ga coupling chemistry (Fig. 7e) compared to physical adsorption (Fig. 7f).

**Figure 8:**
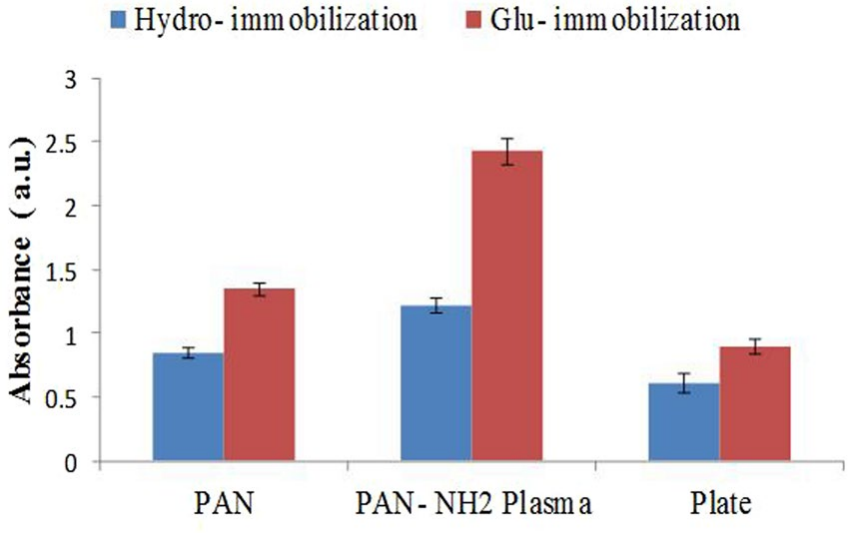
ELISA results obtained from immobilization of Ab through physical interactions and Ga coupling chemistry on different substrates (plasma-treated and non-plasma treated).

The ELISA results of 20 μg/ml Ab immobilization carried out on the conventional 96-well micro-titer plate and plasma-treated and non- treated PAN NFM are shown in Fig. 8. It is observed that, Ab immobilization through Ga coupling chemistry cause sharp enhance in ELISA signal. Moreover, significant enhancement can be also seen on ELISA result of NFM compared to conventional micro-titer plate. We believe that this phenomenon stems from nano-structure of electrospun membranes which provides larger surface area and accordingly higher surface is in access for immobilization of Ab. Therefore, the ELISA signal from PAN NFM was higher than flat conventional micro-titer. Additionally, we found that ammonia plasma treatment has key role on ELISA signal obtained from Ab immobilized sample through Ga coupling chemistry. In our best of knowledge, the creation of amine functional groups by means of ammonia plasma is the prominent parameters on this occurrence. On the other hand, due to creation of hydrophilic functional group, plasma treatment decrease the hydrophobicity of the surface and in this way the Ab immobilization efficacy through physical adsorption will be reduced^11^. Actually, Among different forces that have impact on protein immobilization through physical adsorption on a desire substrate, three of them are known as the most influential driving forces in protein‐surface binding namely, electrostatic (ionic) attraction, hydrophobic interaction and hydrogen (H) bonding^30–32^. It is acceptable that hydrophobic interaction has a major role on protein adsorption and for immobilization of a protein, these forces are in competition with each other.

**Figure 9:**
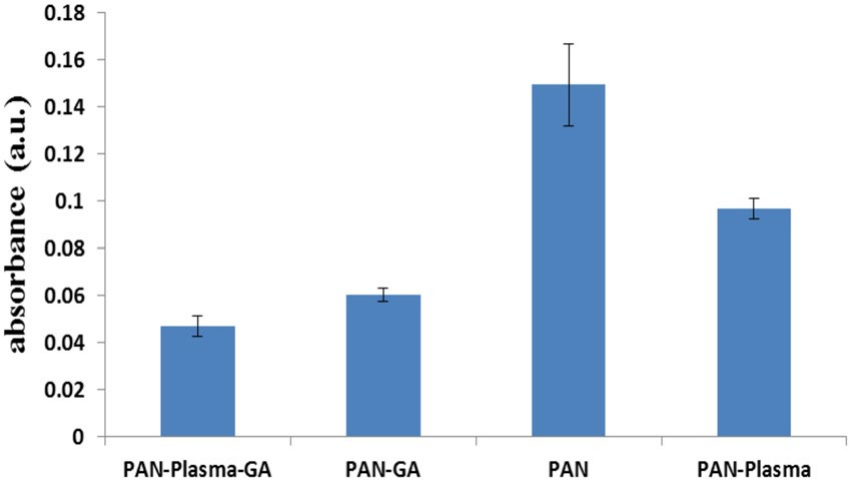
ELIZA results of negative control (samples without incubated primary antibody) for all the groups.

It should be remembered that ELIZA results reported after subtraction of the negative control result of each group. Figure 9 present the ELISA result of negative control (groups which primary antibody is not incubated on the samples) for all the samples. As it is observed, the use of GA as cross-linker causes the amount negative control reduces surprisingly. This phenomenon may due to higher adsorption of BSA as protein to the surface leading to higher efficacy of blocking step and reduction in nonspecific bonding. As a result, using GA not only more antibody can immobilize on the surface, but also more prices results can be obtained in biosensing platforms. Again in consistence with fluorescent microscopy results, it can be observed from Fig. 9 that the highest amount of false answer was obtained for non- plasma PAN NFM due to its hydrophobic nature which increases nonspecific bonding.

**Figure 10:**
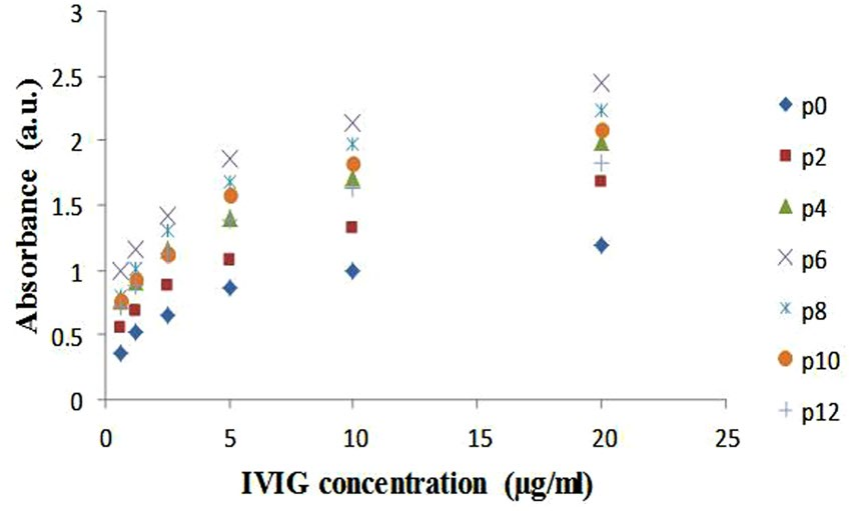
Ab immobilization capacity of differently ammonia plasma- treated PAN NFM.

Ab immobilization capacity of differently ammonia plasma-treated PAN NFM was also investigated. The result showed that with the increase of plasma treatment time, ELISA signal increased indicating higher amount of Ab immobilization occurred on the modified NFM (Fig. 10). Although it was observed the highest amount of amine created on the surface when 8 min plasma implemented on PAN NFM but, surprisingly, it was observed that the ELISA signals were highest for the membrane with 6 min ammonia plasma treatment. It is acceptable that for covalent immobilization of protein on the solid surface, the creation of the desirable functional groups in an optimized concentration is the key parameter for efficient protein immobilization. When there are low number of functional group exist on the surface, the proteins molecules tend to fall on the surface and subsequently lose their activity. On the other hand, high density of functionalized group on the surface can lead to the steric repulsion which in turn cause protein deactivation^33, 34^. Moreover, in an overly functionalized platform, multiple binding between functional groups and individual protein can also lead to the deactivation of the surface and biomolecules. Thus optimal concentration of the surface functional groups permits efficient protein immobilization with serving their functionality for effective binding to analyt molecule. In our case it seems when plasma treatment apply for 6 min, optimum number of amine functional group create on the surface for protein immobilization and higher exposition to plasma lead to steric hindrance and deactivation of protein molecules.

**Figure 11:**
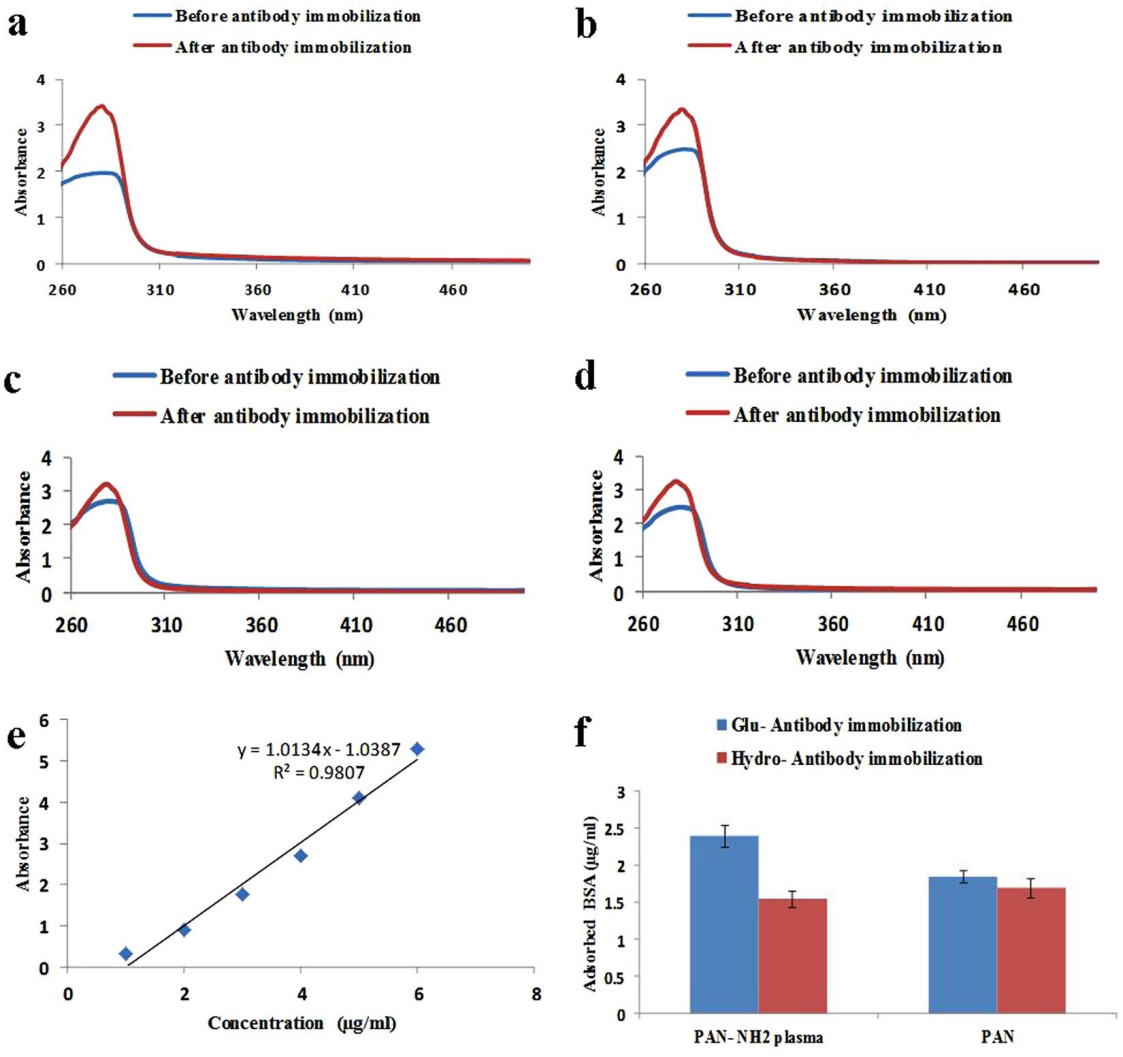
UV–Vis spectra of leached BSA from (**a**) plasma-treated PAN NFM containing Ga coupling agent, (**b**) non-plasma treated PAN NFM containing Ga coupling agent (**c**) plasma-treated PAN NFM without Ga coupling agent, (**d**) non-plasma treated PAN NFM without Ga coupling agent, before Ab immobilization and after Ab immobilization (**e**) Standard calibration curve of BSA (**f**) Calculated amounts of BSA adsorbed from different samples.

Figure 11 represents the differences in the concentration of leached BSA solution from NFM with and without immobilized Ab, which were incubated for 1 h in 3% BSA. These results could help to understand the amount of Ab immobilized to the membrane surface with versatile treatment. The concentration of leached BSA solution was analyzed using UV–Visible (UV–Vis) spectroscopy (Fig. 11a–d). A standard curve for various concentrations of BSA in PBS buffer was also provided and is depicted in Fig. 11e. For clarity, the calculated differences in the concentration of leached BSA from all the samples were illustrated in Fig. 11d. Results of this assessment was again endorsed that higher amount of Ab can immobilize on the surface of plasma-treated PAN NFM through Ga coupling chemistry than physical one.

**Figure 12:**
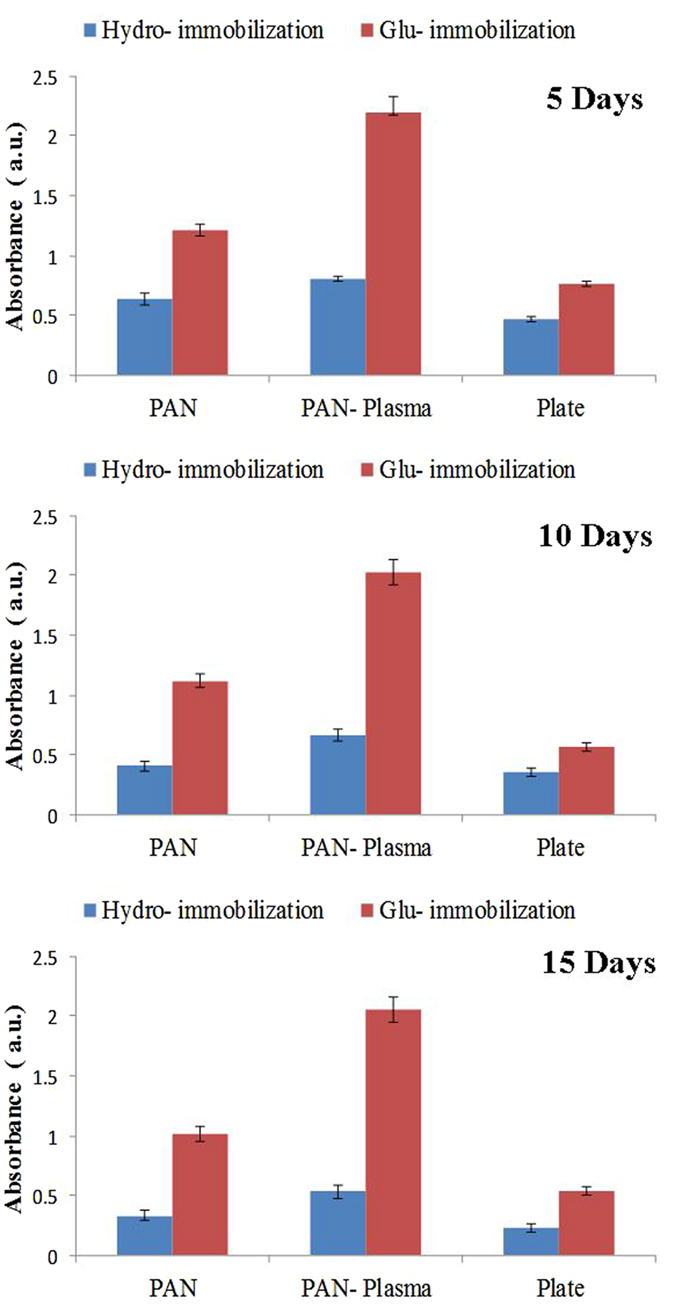
Long-term stability test of Ab immobilization on various substrates through physical interactions and Ga coupling agent. All of samples were stored under dry condition in the presence of silica gel at 4 °C.

As it was mentioned before, conventionally, Ab immobilization on a solid substrate was carried out through physical adsorption. It is well known that physical interactions are unstable and immobilized antibodies tend to deform and loss their functionality^27^. On the other hand, in biosensor platform, vigorous rinsing and ambient condition can reduce the efficiency of operation in turn leading to loss of biosensor sensitivity over time. A stability test was designed to assess the efficacy of Ab immobilization through two different mechanisms on plasma and non-plasma treated PAN membranes. Figure 12 shows the changes in ELISA signal of different substrates after passing desire time of 1, 2 and 3 months of storage in seal condition at 4 °C. As it is observed Ab immobilized samples through Ga serve their functionality over time compared to samples with Ab immobilization physically. This phenomenon indicates powerful effect of covalent bonding to preserve the functionality of proteins over time.

**Figure 13:**
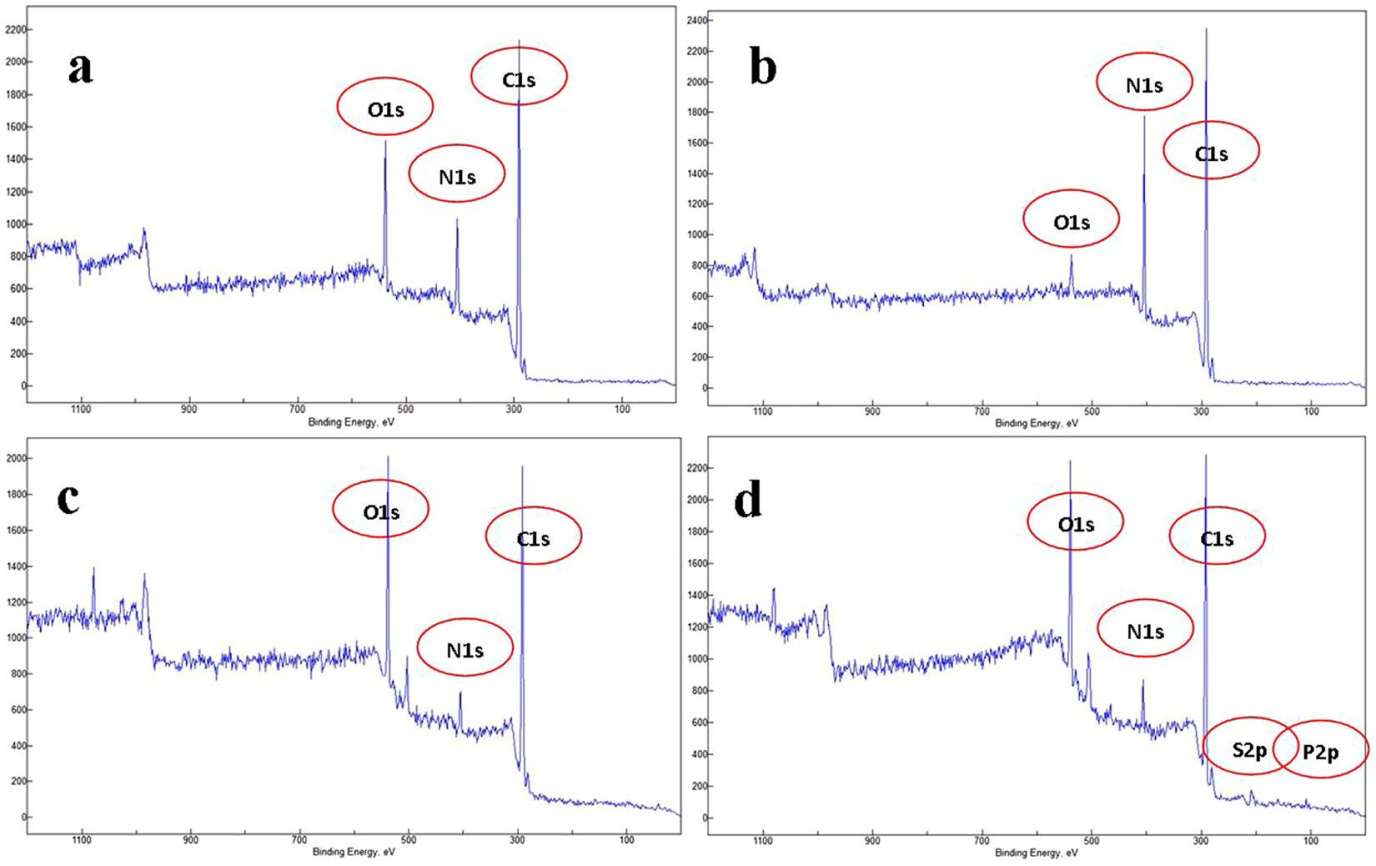
XPS survey spectra of (**a**) PAN NFM (**b**) plasma- treated PAN NFM NFM (**c**) plasma- treated PAN NFM -GA (**d**) plasma- treated PAN NFM -GA-Ab.

**Figure 14:**
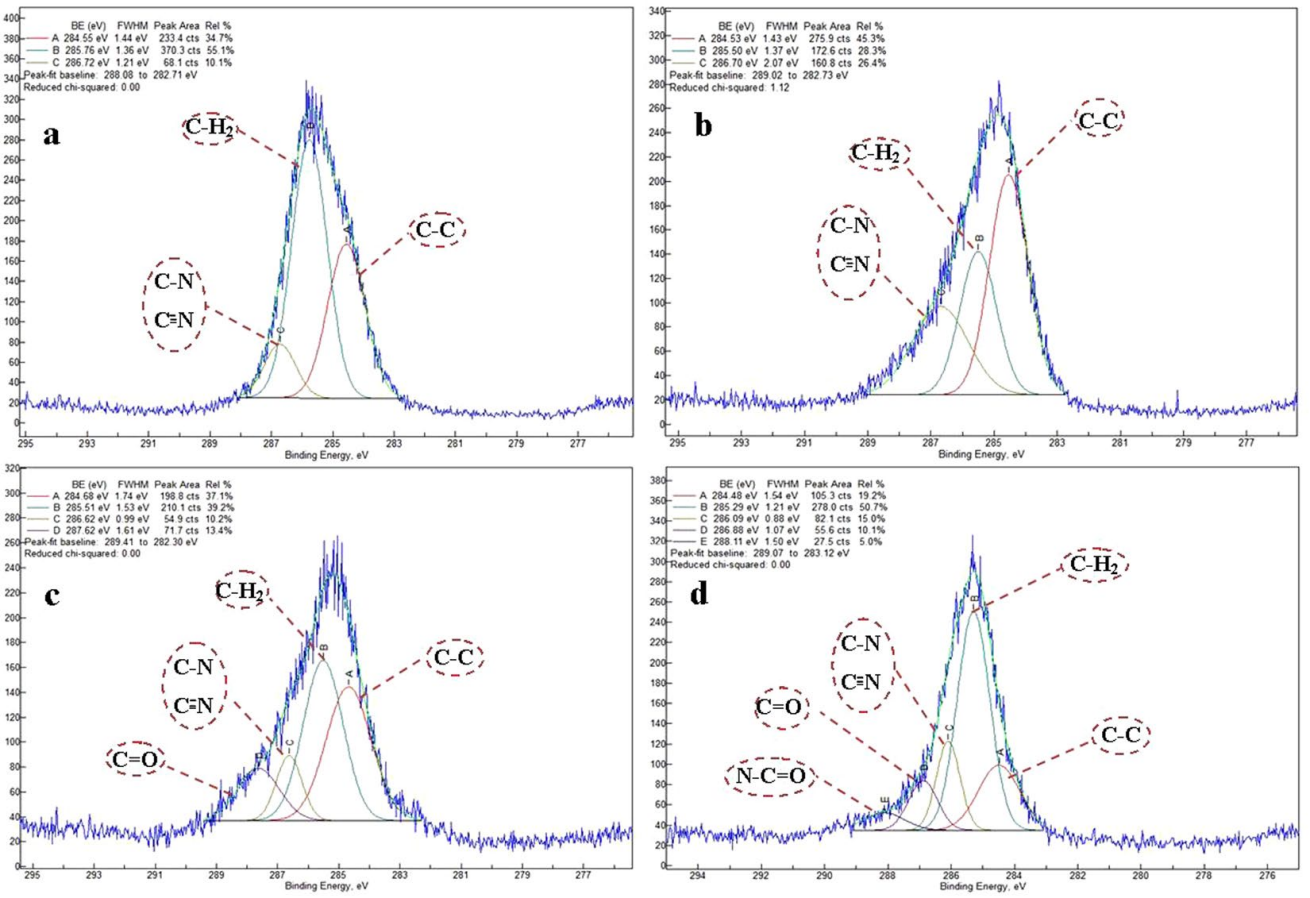
Narrow scan of C 1 s spectra of (**a**) PAN NFM (**b**) plasma- treated PAN NFM NFM (**c**) plasma- treated PAN NFM -GA (**d**) plasma- treated PAN NFM -GA-Ab.

XPS technique has been widely applied to analyze the surface chemical composition of the material before and after different chemical treatment to assess chemical bonding changes occurred on the surface. For this purpose, we exclusively prepared four sets of specimen namely, intact PAN NFM, plasma- treated PAN, GA activated plasma-treated PAN NFM and Ab immobilization on GA activated plasma-treated PAN NFM to support our previous data about formation of covalent bonding between protein and surface through Ga as shown in Figs 13 and 14. The XPS survey scans detected carbon, oxygen and nitrogen in all specimens analyzed. For PAN NFM surface (Fig. 13(a)), C1s, and N1s peaks are observed at 284.7 and 400.2 eV, respectively, as a main constituents of the PAN NFM. The peak at 532.2 eV related to O1s is also observed mainly due to the oxygen contamination on the membrane surface. After plasma treatment, the peak intensity of N1s increases as a sign of successful amination of PAN NFM surface as shown in Fig. 13(b). The incubation of plasma- treated PAN NFM with GA coupling agent causes the peak intensity of O1s increases and N1s decreases indicting the formation of covalent bonding between amine groups of plasma- treated PAN NFM on the surface with one of the aldehyde groups of bifunctional GA (Fig. 13(c)). After Ab immobilization (Fig. 13(d)), S2p and P2p are also appeared on the XPS survey scan as Ab constituents. Thus, the above result indicates that the –NH2 groups are exposed at the surface side of PPAN and Ab molecules successfully were chemisorbed on ammonia plasma- treated PAN NFM through GA cross-linker. Additionally, the peak de-convolution of C1s spectrum regarding the PAN NFM revealed three peaks as shown in Fig. 14(a). The C1s peak at 284.6 eV, 285.5 eV and 286.6 eV were mainly assigned to the C-C and C-H2 and C–N, respectively. As it is observed ammonia plasma treatment clearly increases the peak area percent of C-N chemical bonding as a result of amine group creation on the surface of PAN NFM. After modification of PPAN NFM with GA, a peak at 287.6 eV was appeared which is typical binding energy of C=O related to aldehyde group of Ga. The same observation regarding to GA activation of substrate was also reported previously^35^. Ab immobilization added one another component to the C1s core level spectra of plasma- treated PAN NFM at energy binding of 288.11 eV coming from the amide (HN–C=O) group of the peptide function of Ab^36^. Therefore, XPS analysis clearly revealed that the Ab was successfully coupled covalently on the ammonia plasma- treated PAN NFM surface via GA coupling agent.

Based on our funding, the high capacity of proposed system for the covalent attachment of Ab on the surface of PAN NFM can be utilized in various field of biomedical science including high sensitive surface based immunoassays, tissue engineering and so on.

## Conclusions

Amine group were successfully created on the surface of PAN NFM through ammonia plasma modification. It was found that there are two main mechanisms for amine group creation on the surface of PAN NFM by virtue of ammonia plasma treatment. The first, by reduction of pendent nitrile groups via hydrogen fragment generated through decomposition of ammonia during plasma process and the other by direct incorporation of NH2 fragment exist in plasma environment. 8 min plasma treatment found to be more effective for the generation of the higher amount of amine groups on the PAN NFM surface. But the result of Ab immobilization developed on the plasma-treated PAN NFM through GA coupling agent showed 6 min plasma exposure create optimum functional group for efficient Ab immobilization through Ga. Results also showed the use of ammonia plasma treated PAN NFM for Ab immobilization through Ga coupling chemistry was more promising when compared with conventional physicalally immobilized Ab on PAN NFM. Consequently, our novel and stable Ab immobilized NFM could be used in critical applications including tissue engineering and cell recruitment and homing. Such system could also serve as biosensors in ELISA-like bioassays. The results of our study could serve as a fundamental study for further development in such systems.
